# Pneumatosis Coli Mimicking Colorectal Cancer

**DOI:** 10.1155/2014/428989

**Published:** 2014-10-07

**Authors:** Teresa Jacob, Mohammad Paracha, Marta Penna, Dhili Arul, Jonathan Wilson

**Affiliations:** Department of General Surgery, Whittington Health, Magdala Avenue, London N19 5NF, UK

## Abstract

Pneumatosis coli (PC) is a rare condition of the gastrointestinal tract involving extraluminal gas confined within the bowel wall. We report the case of a 40-year-old gentleman presenting clinically and endoscopically with suspected colorectal cancer. In light of the patient's red flag symptoms, and carpet of polyps seen endoscopically, surgical management by an anterior resection was performed with the patient making a successful recovery. Histological analysis of the resected specimen confirmed pneumatosis coli with no evidence of colonic neoplasia. Although PC can be an incidental finding in asymptomatic patients and considered a benign condition, it can also present as a life-threatening emergency with bowel necrosis and obstruction requiring emergency surgical intervention. Also, when PC mimics malignancy, surgical management is the most appropriate step to ensure that the diagnosis of cancer is not missed.

## 1. Introduction

Pneumatosis coli (PC) or pneumatosis intestinalis is an uncommon condition involving multiple gas filled cysts within the subserosa or submucosa of the gastrointestinal tract wall [1, 2]. The clinical significance of PC ranges from an incidental radiological finding in an asymptomatic patient to a life threatening illness including bowel ischemia and obstruction. PC can be subdivided into primary PC, which occurs in 15–20% of cases and is a benign idiopathic condition, or, secondary PC resulting from other disorders including inflammatory bowel disease, bowel neoplasia, intestinal infections, celiac disease, organ transplantation, and immunosuppression. Patients can present with a wide range of symptoms thus making diagnosis difficult. We present the management of a patient with signs and symptoms suggestive of colorectal carcinoma but ultimately histological diagnosis proved it to be benign PC.

## 2. Case Report

A 40-year-old Afro-Caribbean man presented with a 12-month history of change in bowel habit with alternating constipation and diarrhea. He complained of excessive mucus discharge, abdominal distension, and pain particularly in the left iliac fossa. Notably, he maintained a good appetite and stable weight and denied any rectal bleeding. His only comorbidity was vitiligo, not taking any regular medications, and had no known allergies. There was no family history of colorectal cancer or inflammatory bowel disease.

Months prior to being referred to our colorectal team, he underwent a colonoscopy privately, which showed multiple small polyps in the sigmoid colon. Histopathological results showed “likely hyperplastic polyps with no evidence of dysplasia.”

**Figure 1:**
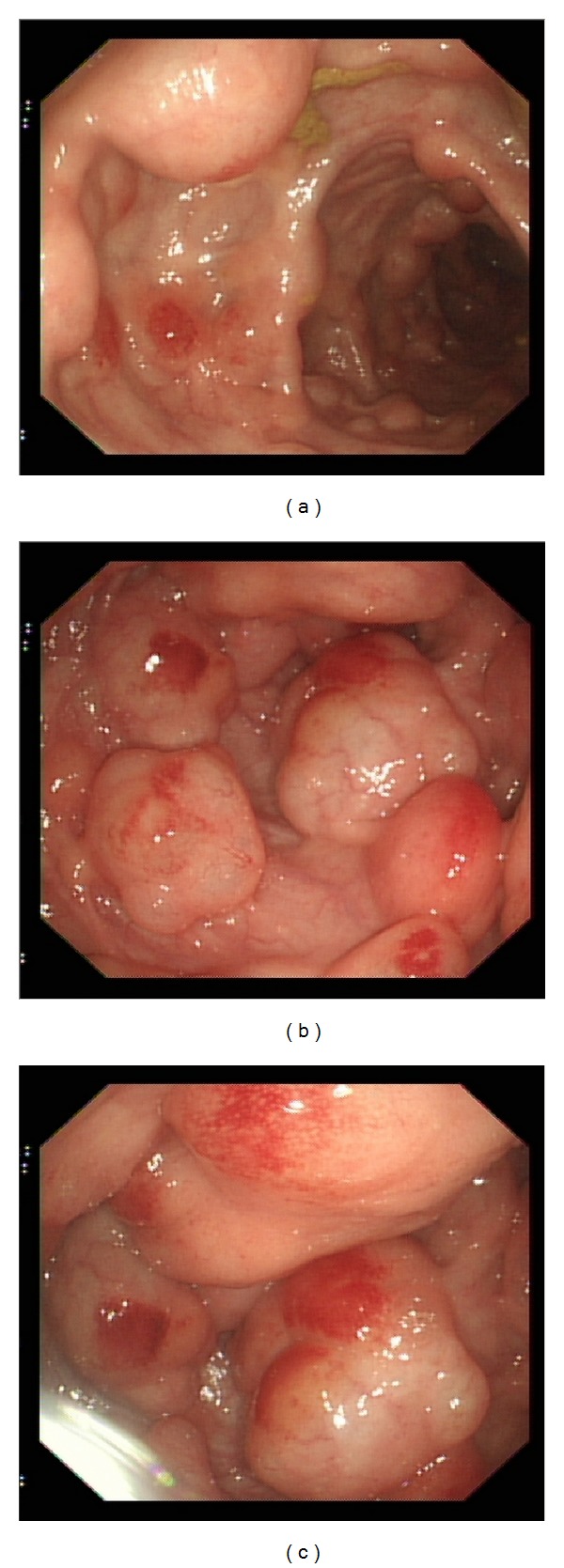
Multiple colonic polyps identified endoscopically within the sigmoid colon.

On review in the outpatient clinic, abdominal and rectal examinations were unremarkable. The blood results were normal including no anaemia. Due to his ongoing unexplained symptoms, a second target colonoscopy was performed and showed a carpet of possible pseudopolyps within the sigmoid colon with normal intervening colon and no evidence of colitis (Figures 1(a), 1(b), and 1(c)). Histology showed “features most in keeping with inflammatory polyps.”

The case was discussed at the lower gastrointestinal cancer multidisciplinary team (MDT) meeting where it was felt that, despite the histological findings, there was sufficient concern endoscopically to merit proceeding to a sigmoid colectomy for definitive diagnosis.

**Figure 2:**
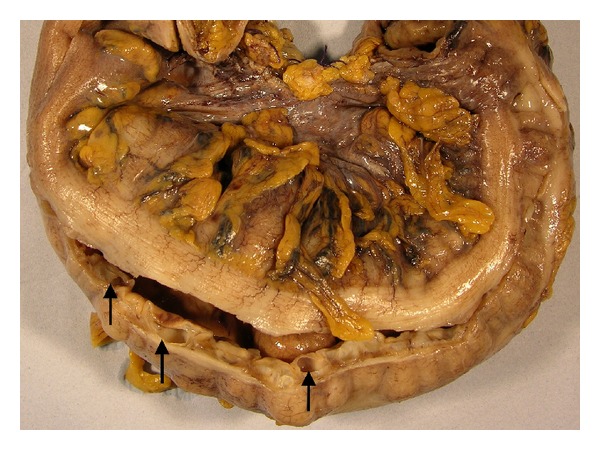
Macroscopic appearance of the resected recto-sigmoid colon. Arrows show numerous air-filled cystic spaces varying in size from few millimeters to more than a centimeter.

**Figure 3:**
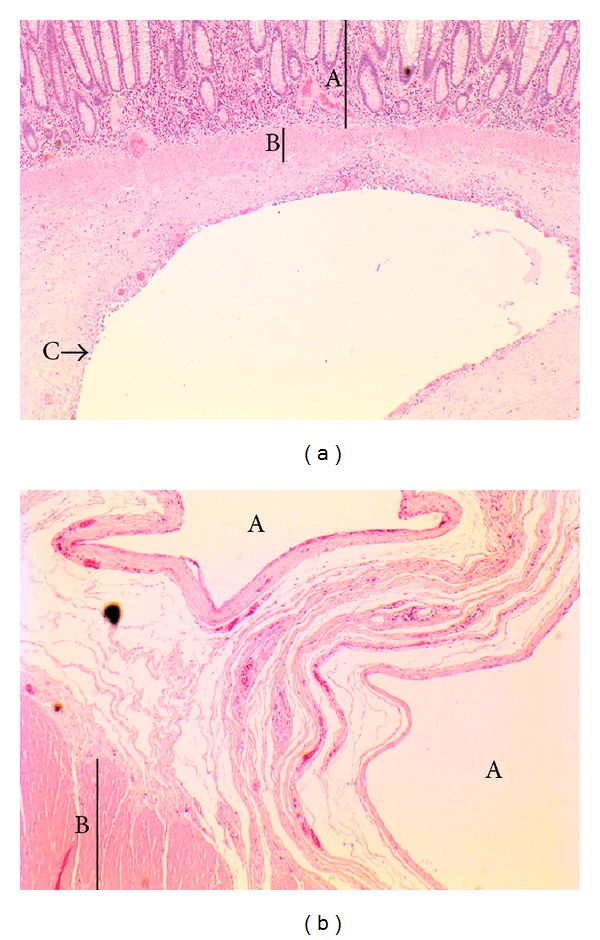
Microscopic appearances of the resected recto-sigmoid colon showing pneumatosis coli. Multiple cyst-like spaces situated predominantly in the submucosa lined by endothelial cells, macrophages, and multinucleate giant cells. Patchy chronic inflammatory cell infiltrate rich in eosinophils in the surrounding stroma. The mucosal layer showed focal hyperplastic changes and patchy nonspecific chronic active inflammation. There was no dysplasia or malignancy. (a) (A) Mucosa, (B) muscularis mucosae, and (C) air-filled space within submucosa. (b) (A) Air-filled spaces, (B) muscularis propria.

The patient underwent an elective laparoscopic anterior resection and loop ileostomy. The procedure was uneventful. The resection was sent for histopathological examination. Macroscopically, the bowel wall showed numerous air-filled cystic spaces varying in size from a few millimeters to more than a centimeter (Figure 2). On microscopy, the cyst-like spaces were situated predominantly in the submucosa and were lined by endothelial cells, macrophages, and multinucleate giant cells. The stroma between the cysts showed patchy chronic inflammatory cell infiltrate rich in eosinophils. The overlying mucosa showed focal hyperplastic changes and patchy nonspecific chronic active inflammation. There was no dysplasia or malignancy. The findings were those of pneumatosis coli with no obvious cause seen on histology (Figures 3(a) and 3(b)).

## 3. Discussion

PC, also known as pneumatosis intestinalis, pneumatosis cystoides intestinalis, and intestinal emphysema, is defined as the presence of gas within the submucosal or subserosal layers of the intestine [1, 2]. Du Vernoy described the first cases of PC in autopsy specimens in the French literature in 1730 [1]. PC was previously reported as occurring most commonly in the small intestine. However, more recent studies have shown that the colon is affected more frequently (46–62% versus 15–27%, resp.), with only 7% of cases affecting both [3, 4]. Further, Wu et al. found that extramural gas was most commonly localized in the submucosal (69.9%) as opposed to the subserosal layer, with 4.6% of cases involving gas in both layers [5]. Symptoms commonly suggestive of PC include abdominal pain, constipation, and bloating, as well as diarrhea, mucous discharge, and rectal bleeding [6, 7].

The etiology of PC may be difficult to ascertain but is often, 85% of cases, due to another underlying pathology, hence termed secondary PC. Precipitating causes of secondary PC include bowel ischaemia, trauma (e.g., surgical or endoscopic), mechanical (e.g., sigmoid volvulus), inflammatory, autoimmune, infectious (e.g., enterocolitis), obstructive pulmonary disorders (e.g., chronic obstructive pulmonary disease and asthma), iatrogenic (e.g., *α*-glucosidase), immunosuppressive, transplantation or neoplasm [8–10]. Five major theories have been proposed as possible mechanisms of pathology. These include mechanical intestinal obstruction or inflammation [11], pulmonary disease leading to pneumomediastinum and gas penetrating into the bowel wall via mesenteric vessels [12], bacterial gas production invading the mucosal barrier [13], chemical/nutritional deficiency theory [14], and iatrogenic PC secondary to chemo- or hormonal therapy [15]. Alternatively, our case may represent the rarer primary PC that has progressed. Little is known regarding the progression of PC and the degree of reversibility.

Radiologically, PC can be identified even on simple plain abdominal radiographs. The cysts usually appear as radiolucent shadows close to the intestinal lumen. If these cysts perforate, then free air can be seen under the diaphragm on an erect chest radiograph. However, the modality of computerised-tomography (CT) has much greater sensitivity and allows assessment of all the intra-abdominal organs [16]. CT therefore becomes particularly useful in cases of diagnostic uncertainty with the endoscopic findings of diffuse colonic polypoid lesions, especially if pneumatosis coli is suspected.

One of the pathognomonic features of PC is pneumoperitoneum without peritoneal irritation as a result of cyst rupture. However, more severe complications occur in about 3% of patients with PC and include pneumoperitoneum, bowel obstruction, volvulus, intussusception, and haemorrhage [17]; all of which are likely to present as an acute emergency and may require surgery. In a prospective review by Knechtle et al. [18], 5 symptoms and signs were predictive of bowel necrosis in patients with PC necessitating surgery:

1. an acute abdomen per history and examination,
2. metabolic acidosis,
3. elevated lactate,
4. elevated serum amylase,
5. presence of portal venous gas.

Conservative approaches have been employed in patients with PC especially when asymptomatic and a benign underlying cause is known. In symptomatic patients with normal biochemical parameters and no evidence of bowel ischaemia or perforation, non-operative management has been shown to be beneficial [19]. Conservative methods include the use of nasogastric decompression, antibiotics and oxygen therapy. A number of reported cases have recommended 70% oxygen inhalational therapy for 5 days or 2.5 atmospheres of hyperbaric oxygen pressure for 150 min/day for 3 consecutive days in order to resolve the gas collection within the bowel wall cysts [10, 19–21].

Diagnosing rare benign conditions can be difficult especially where bowel cancer is part of the differential diagnosis. In such cases, a multidisciplinary approach is essential. Balancing the risks and benefits of either committing to a full oncological surgical resection or adopting a more conservative “surveillance” approach needs to be fully discussed. In our case, the potential risk of cancer and biopsy sampling error was deemed too high to consider expectant management. Colonic biopsies in the presence of multiple polyps are subject to sampling error and hence cannot be fully relied on to exclude cancer. We therefore recommend a cautious approach when managing patients with possible PC in order to avoid missing potential malignancies or delaying treatment until the patient has developed more serious complications necessitating emergency surgery.

## 4. Conclusion

Our case demonstrates that PC may often mimic sinister pathologies in terms of its presentation and endoscopic appearance. Differentiating between benign and malignant disease is not always easy and resection of the affected area may be the best option in order to ensure accurate diagnosis and resolution of symptoms when significant. Primary PC is a benign condition but is also rare, hence the need to exclude other more common bowel pathologies first. PC can also cause serious complications including bowel perforation and obstruction, which may require emergency surgery; therefore, early detection and appropriate management are paramount in providing safe and effective treatment.
